# What triggers explicit awareness in implicit sequence learning? Implications from theories of consciousness

**DOI:** 10.1007/s00426-021-01594-3

**Published:** 2021-09-29

**Authors:** Sarah Esser, Clarissa Lustig, Hilde Haider

**Affiliations:** Department of General Psychology 1, University of Cologne, NRW, Cologne, Germany

## Abstract

This article aims to continue the debate on how explicit, conscious knowledge can arise in an implicit learning situation. We review hitherto existing theoretical views and evaluate their compatibility with two current, successful scientific concepts of consciousness: The Global Workspace Theory and Higher-Order Thought Theories. In this context, we introduce the Unexpected Event Hypothesis (Frensch et al., Attention and implicit learning, John Benjamins Publishing Company, 2003) in an elaborated form and discuss its advantage in explaining the emergence of conscious knowledge in an implicit learning situation.

## Introduction

Implicit learning research is concerned with situations in which individuals learn sequential deterministic or probabilistic contingencies, but lack the intention to learn about these contingencies. Such sequential contingencies might comprise sequential actions (such as learning to blindly type on a keyboard), perceptual events (e.g. learning the sequence of notes in a melody or of visual regularities when driving the same route repeatedly) or complex regularities (e.g. arithmetic principles, Prather, 2012). Empirically, two different tasks are common to investigate implicit learning: The Serial Reaction Time Task (SRTT; Nissen & Bullemer, 1987) and the Artificial Grammar Learning Task (AGL; Reber, 1967). In the SRTT, participants typically respond to sequential stimuli with sequential responses. In general, learning of the underlying sequence is most commonly shown by comparing the reaction times of sequential trials with trials containing a new, unknown sequence. In the AGL, participants learn by observing probabilistic contingencies between strings of, for example, letters or other visual stimuli. Here, learning can, for example, be inferred by showing that participants are able to discriminate strings that follow either the old or new grammar. The hidden regularities in the SRTT and the AGL are usually not mentioned to the participants.

Interestingly, on a subjective level, participants in these paradigms often seem to lack conscious insight that they have learned something. In these cases, it is often presumed that participants acquired unconscious or implicit knowledge. In the simplest sense, when a participant is asked to report the learned sequence at a later time and is unable to do so, is said to have “implicit knowledge”. Participants who can report their knowledge are said to possess “explicit knowledge”. Of course, a large debate is centered around the question of how to determine whether the acquired knowledge in implicit learning paradigms is, in fact, unconscious (see e.g., Newell & Shanks, 2014; Peters & Lau, 2015).

Various objective or subjective measures to determine the conscious or unconscious status of the developed knowledge have been suggested over the years. Objective measures aim to show a performance-based difference between conscious and unconscious knowledge. For instance, process dissociation tasks have been suggested as objective measures (Destrebecqz & Cleeremans, 2001; for critical points see Stahl et al., 2015). These tasks aim to show that unconscious (implicit) knowledge leads to correct responses when the participants are asked to generate the learned sequence on their own (inclusion condition), but also fail to inhibit their acquired sequence knowledge when asked to generate new sequences (exclusion condition). Quite different, subjective measures emphasize the lack of metacognitive knowledge or subjective awareness. While participants with implicit knowledge are often able to predict the next correct response or stimulus, they express subjective uncertainty. For example, they put low instead of high wagers on the correctness of their responses. By contrast, participants with conscious (explicit) knowledge express high certainty on correct responses but low certainty on wrong responses (Dienes & Seth, 2010; Haider et al., 2011; Persaud et al., 2007). Thus, there are conceptual differences when assessing the status of knowledge acquired in an implicit learning situation. Furthermore, presupposed that implicit learning tasks can lead to implicit, unconscious knowledge, there usually are participants who develop explicit, consciously accessible knowledge. Based on these findings, we will adapt the term “implicit knowledge” for subjectively and behaviorally unconscious knowledge and “explicit knowledge” for conscious knowledge.

This difference in conscious availability of the acquired knowledge suggests that implicit learning settings could provide a promising opportunity to investigate how implicit or explicit knowledge is related to the representational status of the acquired knowledge and which mechanism mediates between implicit and explicit knowledge. Is explicit knowledge based on the same acquired representation as implicit knowledge? If so, does the same learning mechanism change the representational quality (e.g. its strength is increased over the learning period) which is then accompanied by a gradual change in conscious accessibility? Or does the conscious status depend on a different access to that representation? A different possibility would be that implicit knowledge is based on different representations than explicit knowledge. These latter two options would imply that an “implicit learning” process leads to strictly implicit knowledge, while explicit knowledge requires an additional process that grants conscious accessibility.

This leads to a close connection between implicit learning research and general theories of consciousness which aim to explain how consciousness originates from unconscious processes. This way, implicit sequence learning can enrich theoretical views on consciousness with new experimental approaches and help to test predictions, thereby, improving current weaknesses or identifying unexpected problems of the respective theories. Likewise, the question how explicit knowledge arises from an implicit learning situation can profit from testing predictions from general theories of consciousness.

The central aim of this article is thus to discuss two current models in implicit learning research that aim to explain how consciously accessible, explicit knowledge can develop from implicit learning situations and to assess their correspondence with current theories on consciousness. One of these views proposes that explicit knowledge depends on the strengthening of associative links (i.e., the representational quality). In this case, it is assumed that conscious knowledge will develop gradually, either by strengthening single transitions (Cleeremans, 2008, 2011; Cleeremans & Jiménez, 2002) or by integrating single elements into sequence chunks (Perruchet & Vinter, 2002; Perruchet et al., 2002). On the other hand, there are theories that propose an indirect link between implicit and explicit knowledge. One of these theories is called the *Unexpected Event Hypothesis* (Frensch et al., 2003). From this theoretical viewpoint, unconscious (implicit) knowledge and conscious (explicit) knowledge are based on different representations. Implicit knowledge relies on representations acquired during learning that lead to behavioral changes (e.g. faster RTs). Explicit knowledge develops by detecting mismatches between expected and experienced behavior. If such a mismatch is detected a second (and subjectively explicit) inferential process is triggered that can lead to the discovery of sequential contingencies inherent in the task. In this case, a new representation of the sequential contingencies is acquired, which is independent of the implicitly learned representation. This new representation is explicitly accessible. Thus, explicit knowledge is not assumed to develop gradually from implicit knowledge, as is presumed by strengthening theories, but instead in an abrupt change from an unconscious into a conscious knowledge state.

We will review current evidence for each of these theoretical classes. Furthermore, these two viewpoints and their empirical evidence will be reconciled with two prominent theories of consciousness: One is the *Global Workspace Theory* (Baars, 1997, 2005), the other is the *Higher-Order Thought Theory* (Lau, 2008; Rosenthal, 1997, 2012). We will discuss the advantages and disadvantages of each perspective and their implications for the development of explicit knowledge in implicit learning situations.

## The serial reaction time task (SRTT) and its relevance for consciousness research

In this section, we will briefly outline how implicit learning paradigms can provide an important opportunity to investigate the connection between unconscious and conscious processes. As we have briefly stated in the introduction, there are various implicit learning paradigms which all have in common that a sequence of responses or stimuli is learned without the intention to do so. Here, we will mainly focus on the SRTT, as we believe that its simple, yet flexible structure makes it a particularly interesting instrument to investigate how implicitly acquired representations can become consciously accessible.

The SRTT has the potential to provide both, online and offline measures of learning. During the training part, in which participants are trained with a sequence of stimuli or responses, error rates and reaction times can be utilized to assess learning. Subsequent tests, such as the previously described Inclusion/Exclusion Task or the Wagering Task, are usually employed to determine whether or to what extent the acquired knowledge is implicit or explicit. However, the training part of the task can also have the potential to detect whether knowledge is explicit. Moreover, it might also have the potential to confine the time frame in which a qualitative change from an implicit to an explicit representation of the learned sequence occurs. It has been found that participants who develop explicit knowledge in a SRTT often show a sudden decrease in reaction times in the training phase. This so-called RT-drop indicates that participants change their behavior qualitatively (Haider & Frensch, 2005; Haider et al., 2005; Haider & Rose, 2007; Lustig et al., 2021; Rose et al., 2010; Wessel et al., 2012) and do not mandatorily need to process the stimulus anymore to perform the task. This stimulus-independency of explicit sequence knowledge is further supported by studies that show that participants are no longer affected by incongruent stimulus–response characteristics, such as the Stroop Effect (Haider et al., 2011) and the Simon Effect (Koch, 2007), or by a frequency-induced response bias (Tubau et al., 2007). Thus, analyzing reaction times in a SRTT has the potential to classify participants with explicit sequence knowledge online during training, as well as potentially grasping the point in time when explicit knowledge occurs (Rose et al., 2010).

From this perspective, the SRTT is a useful addition to the investigation of qualitative differences between unconsciously and consciously accessible representations, as well as the mechanisms that mediate between them. Mostly, such questions are approached by priming studies (e.g., Del Cul et al., 2009; Kouider & Faivre, 2017; Kouider et al., 2010; Lau & Rosenthal, 2011; Overgaard, 2003). Priming studies provide a well-controlled opportunity to study the differences between conscious and unconscious processing on a trial-by-trial basis. In priming studies, unconscious processing is operationalized either by weak signal strength or by inattention towards the critical stimuli or stimuli features (Dehaene et al., 2006). The advantage of implicit learning paradigms, however, is that they provide an opportunity to create situations where signal strength can be controlled in a wider range (e.g., by controlling training duration or sequence complexity) while the resulting representations have a high temporal stability (Tamayo & Frensch, 2015). At the same time, the role of attention towards the sequence can be investigated, with evidence so far suggesting that selective attention (i.e., having participants respond to the sequential feature vs. responding to a sequence-irrelevant feature) towards the sequence does not lead to more conscious knowledge but might be a prerequisite for implicit learning (Jiménez & Méndez, 1999; Turk-Browne et al., 2005). A further difference between priming and implicit learning paradigms is that the former can provide insight how certain singular simple or complex (most often visual) percepts which already have an existing entry in long-term memory can become conscious. By contrast, implicit learning studies can investigate how whole *newly acquired* knowledge structures about relations between clearly perceivable events in the environment can become conscious. Implicit learning can hereby help to investigate how the cognitive system can learn about its own internal states; how the system changes from a state of not knowing that the internal knowledge base has changed to knowing that new knowledge has been developed and that current consciously accessible believes about states in the world need to be adjusted or replaced. Thus, implicit learning research could also be seen as an interesting link to research on insight and creativity in which it is often proposed that creative ideas result from or, at least, are largely supported by consciously inaccessible processes (Dietrich & Haider, 2017; Fedor et al., 2017).

The term creative “insight” implies that there is a very brief moment in time in which the cognitive system changes its state from consciously not knowing to knowing. Nevertheless, it is important to see that research on insight and creativity often stresses that such apparently sudden “insight” moments are preceded by other events, including the moment of noticing that there is a problem that needs to be solved over an incubation period (spanning from years to minutes) until finally coming up with a new idea (Cosmelli & Preiss, 2014; Dietrich & Haider, 2017; Hélie & Sun, 2010). Thus, there might be different, hierarchical representational contents an individual can become aware of Kouider et al., (2010). In an implicit learning task, this can encompass consciously noticing that there is something about the task that has not yet been recognized before, noticing that certain events (finger movements, stimuli, etc.) appear systematically, to lastly discovering the actual sequence that governs the task. However, regardless of the specific content, these changes in awareness could result from a sudden or a gradual change in the underlying representations. While the SRTT has the potential to explore various aspects of these insight stages, what might be of particular interest in the light of the Unexpected Event Hypothesis is the moment where an individual notices that their consciously held beliefs about the task do not match their perceived experiences, which could trigger an inferential process that finally leads to recognizing the sequence in the task.

## How to conceptualize the transition from implicit to explicit sequence knowledge

In the following, we will briefly outline two theoretical viewpoints about the development of explicit knowledge in an implicit learning situation. Afterwards, two prominent theoretical perspectives on consciousness will be introduced. These are the *Global Workspace Theory* (Baars, 1997; Baars & Franklin, 2003; Dehaene & Changeux, 2011; Dehaene & Naccache, 2001) and the *Higher-Order Thought Theory* (Dienes & Perner, 1999; Lau & Rosenthal, 2011; Rosenthal, 2012; see Dehaene et al., 2017, or Shea & Frith, 2019, for an argument why both, the Global Workspace Theory and the Higher-Order Thought Theory, are important for consciousness studies). We will elaborate how both theoretical views have already been applied in the field of implicit learning and how they fit the current theoretical views and empirical data on the transition from implicit to explicit knowledge. We will try to illustrate which problems both perspectives have, when they are used to explain how explicit knowledge arises from an implicit learning situation. Finally, we aim to discuss in which direction future research could go to tackle these issues.

## Theoretical views on the development of conscious knowledge in implicit learning situations

As briefly stated in the introduction, there are two distinct theoretical perspectives on how explicit knowledge can develop in an implicit learning situation. One proposes that there is a continuous transition from an unconscious to a conscious state, while the other assumes that knowledge is either implicit or explicit and that the transition happens in a sudden representational change.

The idea of a gradual change in representational quality goes back to Cleeremans and Jiménez (2002). They proposed three different factors which influence the quality of a representation: (1*) Stability*, i.e., the time a certain activation pattern can be maintained, (2) *strength*, i.e., the number of modules involved and their respective activation strength, and (3) *distinctiveness*, i.e., the extent of overlap between representations within a functional network (see Kinsbourne, 1996, for a similar position). While implicit learning first leads to very weak representations, with practice these representations gradually gain quality and can result in explicit knowledge. This proposal has later been elaborated further in the *Radical Plasticity Hypothesis* by Cleeremans (2008, 2011, 2014) by adding a hierarchically higher, second-order learning system. The lower-order or first-order learning system develops implicit knowledge through interaction with the environment. This knowledge is never conscious; it is labeled as knowledge *within* the system. For consciousness to arise, the first-order information needs to be redescribed as a meta-representation; that is, knowledge *for* the system (Clark & Karmiloff-Smith, 1993). The first-order representation itself becomes an object of a representation for higher-order systems. This higher-order system receives input from the first-order systems and learns that the state of the first-order system has changed as it becomes more accurate and thereby develops a higher-order attitude towards the first-order knowledge (e.g. “knowing that …”, “hoping that …”, “believing that …”). This higher-order representation is assumed to be a new representation involving a broad pattern of activation over different processing units which is only indirectly shaped by the changes of the connection-weights within the first-order system. The proposed learning mechanism behind the first- and the higher-order learning system is the same; both systems gradually improve the quality of a representation with each learning trial. Thus, the higher-order representation gradually becomes more consciously accessible, as it changes from “not knowing” to “knowing”.

The other theoretical account, the Unexpected-Event Hypothesis, agrees that it is not the implicit knowledge that becomes conscious itself. Rather, a second learning mechanism leads to the acquisition of explicitly accessible knowledge. However, this second mechanism does not lead to a gradual development of conscious access. The crucial idea of the Unexpected Event Theory is that explicit sequence knowledge can only develop when an individual unexpectedly notices a change in their own behavior. In an implicit learning situation, the interaction with the task leads to a continuous improvement of the responses to the stimuli; they become more accurate and faster. It can be this improvement or, for example, the feeling that the task becomes more fluent or easy, that there is a certain rhythm in one’s own responses, or an external event that can trigger an intentional search for the sequence. Generally speaking, the Unexpected Event Theory involves a monitoring process which constantly compares observable expected and actual behavior. This comprises internal, subjectively experienced, as well as externally observable behavioral deviations from one’s expectations.

For example, giving the (correct) response before the stimulus was shown could clearly be a surprising event for the participant. Haider and Frensch (2009) varied the RSI to manipulate the opportunity for participants to emit a response before the next stimulus occurred. They found more explicit sequence knowledge when the task allowed participants to emit premature responses. Likewise, Rünger and Frensch (2008) demonstrated that exchanging the trained sequence with a new sequence led to more explicit knowledge, than the same amount of training with only one sequence. The authors attributed this effect to the participants noticing surprisingly slower responses and attributing them to a change in the structure of the task. Controlling for the number of sequential and non-sequential trials, Esser and Haider (2017) showed that arranging regular, sequential and irregular, random trials in mini-blocks led to more explicit sequence knowledge, compared to a condition where regular and irregular trials were mixed randomly. In this case, the authors attributed this effect to providing the participants the opportunity to experience systematic differences in the fluency of the task.

Under the Unexpected Event Hypothesis, these consciously perceivable differences in the expected and observed behavior are critical for recognizing that the own knowledge is different from the expected knowledge. This does not imply that the individual instantly also gains insight into the exact sequence structure. Instead, the detected conflict triggers an attributional process to adjust its predictions and reestablish coherence between the distal environment and one’s proximal model of it. Comparable monitoring-models have been established in neurocognitive models of conflict-detection and adaption (Botvinick, 2007; Botvinick et al., 2001), metacognitive control (Koriat, 2000, 2012, 2015), or memory (Whittlesea, 2002; Whittlesea & Williams, 2000). Thus, the triggered attributional processes do not necessarily lead to explicit sequence knowledge, if another explanation seems more likely to account for the unexpected experience (see also Reisenzein et al., 2019). Haider and Frensch (2005) have shown that premature responses in an implicit learning task did not result in more explicit knowledge if another, simpler explanation for these responses was provided (e.g. “attentional lapses”). Importantly, the secondary explicit learning process is not assumed to directly have access to the implicit knowledge. Instead, the explicit learning process will result in a new representation based on the available information. In this context, Schwager et al. (2012) have shown that after perceiving an unexpected event, participants will learn a new sequence just as well as the sequence they were trained with.

A suggestion that comprises aspects of both perspectives, the Radical Plasticity and the Unexpected Event Hypothesis, has been made by Scott and Dienes (2008, 2010). They suggested that implicit learning influences familiarity judgements, which enable individuals to correctly distinguish strings that follow the implicitly learned structure from those that do not. With repeated familiarity judgements, the individual will learn that their judgements are correct and thus explicit *judgmental knowledge* (knowing that one knows) develops gradually. However, to acquire explicit *structural knowledge* (knowing the exact structure of the implicitly learned information), a second explicit learning mechanism is required that tests different hypotheses about the reason for the correct judgements. When and how this explicit learning mechanism is triggered remains unclear.

In the following sections, we briefly describe the main aspects of the Global Workspace Theory and The Higher-Order Thought Theory as well as their relation to both, the Radical Plasticity and the Unexpected Event Hypothesis.

### Global Workspace Theory

The Global Workspace Theory is a prominent functional and neuroscientific theory of consciousness. The basic assumption of the Global Workspace Theory is that the brain contains a multitude of functionally highly specialized subsystems working in parallel. Information in these subsystems is unconscious, there is no phenomenal- (Block, 2007; Chalmers, 1995), micro-consciousness (Lamme, 2006), or anything alike associated with information processing in these networks. Per se, these networks work encapsulated, that means they exchange information only within hard-wired or acquired pathways to fulfill their specialized task. This encapsulation enables the brain to handle a massive amount of input in parallel (Baars, 1997). Nevertheless, coherent interaction with the environment requires serial output and therefore a mechanism is needed that selects the most relevant information. Here, the theory postulates a global workspace mechanism which provides the necessary infrastructure, neurologically mainly realized by thalamo-cortical long-distance neurons of the prefrontal and the anterior cingular cortices (see Baars et al., 2013 for a detailed elaboration of the neuronal architecture). The global workspace is able to select relevant information, to prevent interference, to allow the encapsulated modules to exchange information, and to flexibly establish temporary networks between these modules (Dehaene & Naccache, 2001).

The Global Workspace Theory uses a blackboard metaphor for describing how the global workspace works. When a module gets selected to enter the global workspace, it can broadcast its content to any other network in the brain. Other modules can access the information on the blackboard and process it in their specified function. The information from the broadcasted module is no longer encapsulated. It is now said to be amodal because it is no longer bound to the specialized processes of the module it originated from. Instead, it is now processed in a broad context of unconscious subsystems. These subsystems include, for example, perception, language, intentions, self-concepts, expectations, memory, and exclusive access to working-memory functions (Baars, 1997, 2005; Baars & Franklin, 2003; Baars et al., 2013; Cowan, 2010; Persuh et al., 2018; Schwager & Hagendorf, 2009). Thus, the Global Workspace Theory avoids the assumption that there is a certain instance where consciousness is “created” or that consciousness is an additional phenomenal quality that accompanies certain processes. Instead, consciousness is functionally defined by the global accessibility of representations and the resulting enabling of behavioral consequences.

Crucial to the Global Workspace Theory as a functionalist theory of consciousness is that conscious processing is equalized with the global accessibility of information and the thereby enabled options of processing this information. Neuroimaging shows that this de-capsulation of information is accompanied by a neurological “ignition”, a sudden, strong activation of a vast variety of cortical and subcortical regions (Dehaene & Changeux, 2011; Dehaene & Naccache, 2001; Del Cul et al., 2009; Rose et al., 2010; Schuck et al., 2015; Wessel et al., 2012). Hence, in the Global Workspace Theory the transition from unconscious to conscious processing is seen as an all-or-none phenomenon; there is no gradual consciousness. What may differ, however, is the level of representation that gains access to the global workspace. The levels of representation can vary from low (e.g. simple shapes or levels of intensity) to high (e.g. whole object, words, meaning), which all can be accessed independently and thus account for varying levels of the quality of conscious representations (Kouider et al., 2010).

To explain how a representation can change from an unconscious to a conscious state, while at the same time avoiding to assume that some instance “knows” (i.e., a homunculus) which information to select, the Global Workspace Theory suggests a stochastic bottom-up variation-selection mechanism for explaining how the most relevant information is selected from the enormous amount of unconscious information (“Neural Darwinism”, Changeux & Dehaene, 1989). Every unconscious module constantly competes for access to the global workspace (variation component), while the global workspace sets a selection function depending on current goal states. Only one module or coalition of modules will show the strongest activation in the context of the current goal-state-dependent content of the global workspace and will therefore win the competition for global broadcasting (“winner-takes-it all”, Shanahan & Baars, 2005). If a bottom-up signal surpasses a certain threshold, it is assumed to receive top-down amplification to remain maintained (it is said to receive “attentional amplification”). Thus, Dehaene et al. (2006) propose a 2 × 2 taxonomy in which an unconscious representation can have (a) strong or weak signal strength and, independent from signal strength, (b) can or cannot be amplified by top-down attention. Only when both criteria are given, signal strength is high and top-down attention towards the unconscious content is provided, the sufficient conditions for consciousness are met.

### Global Workspace Theory and the emergence of conscious knowledge in implicit learning

What are the implications of the Global Workspace Theory for implicit learning research and the explanation how explicit knowledge develops in an implicit learning situation? A first prediction is that the change from an unconscious to a conscious state happens in a sudden “insight” that a sequence has been learned, instead of a gradual change towards consciously accessible knowledge (Marti & Dehaene, 2017). There is some empirical evidence that, in fact, the transition from implicit to explicit knowledge is reflected in a sudden representational change. These studies aim to examine the point in time where an individual becomes able to verbalize their acquired knowledge or use it strategically. For example, Haider et al. (2011) provided evidence that most of the participants who showed a sudden drop in their RT during learning were able to verbalize their knowledge by the end of training. The RT-drop seemingly reflects the moment where participants switched from stimulus- to plan-driven control (Tubau et al., 2007). Moreover, neuroimaging data showed that a sudden coupling of gamma-band activity and increases of the BOLD-signal in the ventrolateral prefrontal cortex, the medial and ventrolateral prefrontal cortex and the ventral striatum preceded such an RT-drop, respectively strategy change (Lawson et al., 2017; Rose et al., 2010; Schuck et al., 2015; Wessel et al., 2012). These changes might reflect the sudden “ignition” of cortical activity which, as postulated by the Global Workspace Theory, accompanies the transition from an unconscious to a conscious state (Dehaene & Changeux, 2011; Dehaene & Naccache, 2001).

While such results implicate that conscious insight into implicitly learned representations seem to happen rather sudden instead of gradually, they do not provide information about how and why these transitions occur. A non-linear transition can occur due to an underlying slow gradual learning processes as well as through a spontaneously triggered, inferential explicit learning process. To explain how an unconscious, implicit representation can become consciously accessible under the Global Workspace Theory, two essential aspects need to be clarified: first, it needs to be explained how implicit, encapsulated information can reach a representational strength high enough to win the competition for access to the global workspace. Second, it needs to be explained how a goal state arises that provides the necessary top-down amplification for the implicit information.

Certainly, with ongoing practice in an SRTT, the representational strength or quality will gradually increase. The Global Workspace Theory allows the proposal that with enough practice, the representational strength or quality could be high enough by itself to win the competition and enter the global workspace (Cleeremans & Jiménez, 2002). This is what happens, when a signal with very high bottom-up strength is presented (e.g. a loud noise). However, it seems rather unlikely that an implicit learning process results in a signal that is strong enough by itself to win this competition without any additional top-down amplification. Likewise, a gradual higher-order learning process, as it is suggested by Cleeremans (2008, 2011), would, in the light of the Global Workspace Theory, require an additional explanation when and how the higher-order learning process changes from a gradual increase in representational quality to a non-linear increase in activation that corresponds with the sudden ignition proposed by the Global Workspace Theory. Rather, it seems that an explanation is needed how the system gets into a state in which the encapsulated module containing implicit information provides the fitting information to the selection function set by the global workspace and thus will receive additional top-down amplification.

Here, the Unexpected Event Theory provides a simple explanation: A monitoring process can detect a mismatch between expected and experienced performance. Because the mismatch is subjectively perceivable in one’s own behavioral output or in internally perceived aspects of the task, this mismatch results in a new state of the global workspace that sets a fitting selective function for the implicitly learned representation.

Whatever the mechanism is that triggers the global workspace to allocate top-down amplification to the implicitly learned representation, it is furthermore important to ask, whether it is the implicit representation itself that will become a conscious representation or whether a new explicit representation will develop.

The Unexpected Event Theory suggests that the latter is the case; a detected mismatch only triggers a conscious attributional process with the purpose of finding any explanation for this mismatch. If an explanation different from an underlying sequence is more likely to the participant, the (sequence) knowledge remains implicit (Haider & Frensch, 2005; Wilbert & Haider, 2012). If, however, it seems likely to the participant that an underlying sequence is a reason for their behavior, a new, explicit learning process will learn the sequence, fully independent of the implicitly learned representation (see Schwager et al., 2012).

To sum up, when the Global Workspace Theory should be applied to explaining a transition from implicit to explicit knowledge two questions need more investigation in the future: first, how does the global workspace get into a state that can provide top-down amplification to the implicitly learned representation? Second, is it the implicit information that becomes conscious itself once it is selected or does the content of the global workspace only mobilize a second, explicit learning process?

### Higher-Order Thought Theory

While the Global Workspace Theory is a specific theory of consciousness, Higher-Order Thought Theories are an umbrella term for a wider range of theories, which are concerned with the metacognitive aspects of consciousness (Lau & Rosenthal, 2011). Here, we focus on a Higher-Order Thought Theory that goes back to the work of Rosenthal (1997; Dienes & Perner, 1999). In its core, it differentiates between first-order and second-order (or higher-order) states. First-order states refer to simple input–output rules of any sensory or motor system. This can be understood in analogy to the parallel working modules in the Global Workspace Theory. Encapsulated, respectively implicitly learned information can be seen as a first-order state which is unconscious. Not only the human brain, but any simple or complex machine, which shows discriminatory performance, has first-order states (e.g. perceiving light of a certain wavelength results in the output of detecting red).

Consciousness, according to Higher-Order Thought Theories, crucially depends on developing higher-order knowledge about this first-order knowledge. Put simply, consciousness means knowing that one knows. This comprises the ability for self-reflection, self-reference and a propositional attitude (e.g. “I *know/believe/guess* that it is red that I see”, “It is *I*, who sees red”, “it *is red* that I see”, Dienes & Perner, 1999). What is needed for consciousness is a mechanism that allows the brain to draw inferences about its own internal first-order states and about how these relate to states in the environment. Different theoretical suggestions and models have been put forward to describe the learning process behind the acquisition of higher-order knowledge about first-order states (Fleming & Daw, 2017; Lau, 2008; Lau & Rosenthal, 2011).

### Higher-Order Thought Theory and the emergence of conscious knowledge in implicit learning

The theoretical view put forward by Cleeremans (2008, 2011, 2014) clearly applies Higher-Order Thought Theories to the question how explicit knowledge develops in an implicit learning situation: through interaction with the environment, a first-order representation is developed and is gradually improving in quality. The higher-order system receives input from the first-order systems and learns that the state in the first-order system has changed and thereby develops a higher-order attitude towards the first-order knowledge (e.g. “knowing that …”, “hoping that …”, “seeing that …”). This higher-order knowledge is not per se conscious but can become conscious, once its representational quality is strong enough. A very valuable aspect of this theory is that it connects well-established connectionist learning theories with the development of consciousness.

Pasquali et al. (2010) have investigated the relation between first-order sensitivity and higher-order awareness in simulations of neural networks within different paradigms (i.e. Blindsight, Iowa Gabling Task, and an AGL Task). These results supported the assumption that the higher-order representations gradually improve with the learning progress of the first-order system. Using the Post-Decision Wagering Task (Persaud et al., 2007), the authors showed that the higher-order networks gradually changed from classifying random answers as being correct to classifying only correct responses as being correct (by giving high wagers on correct responses).

There are debates on how exactly the relation between first-order knowledge and a meta-cognitive learning mechanism should be modeled, with most of the suggested models being based on bottom-up signal-detection theories (Barrett et al., 2013; Fleming & Lau, 2014; Maniscalco & Lau, 2012, 2016). What these models have in common is the gradual development of higher-order knowledge. The conscious state of a representation changes from guessing, which represents being unconscious about a first-order representation, to knowing, which represents being conscious about a first-order representation (Dienes & Scott, 2005; Sandberg et al., 2010).

A rather simple higher-order learning mechanism, as proposed by Cleeremans (2014), might indeed provide an important basis for a cognitive system to determine what first order-state it currently is in. The assumption that a meta-cognitive learning mechanism plays a significant role in gaining conscious insight into otherwise unconscious information processing is very promising, as it describes the brain’s ability to learn not only about external information but also about its internal states. However, there are a few open points that should be considered in future research.

The higher-order learning process informs the system that knowledge has been acquired, but knowing that one knows (instead of guessing) the correct response is not equal to knowing that there is an underlying sequence or even knowing what exact rules constitute this sequence (Scott & Dienes, 2010). It could be argued that under Higher-Order Thought Theories this aspect is less important than under the Global Workspace Theory, because consciousness is defined as possessing a higher-order representation of the first-order contents. Further functional properties, such as being able to verbalize the sequence, or being able to flexibly transfer this knowledge to new, different situations are of less importance. However, the question remains: even if consciousness relies on gradual metacognitive learning processes, how is that learning mechanism connected to explicit knowledge of the underlying sequence?

Another question to ask is if correctness of the first-order performance is the only or most important target of higher-order learning systems. Knowing that one knows might not only rely on assessing the correctness of the behavioral output. For example, noticing premature responses before the next stimulus occurs (Haider & Frensch, 2009), sudden changes in the sequential structure which lead to slower reaction times (Rünger & Frensch, 2008), or changes in the perceived fluency of the task performance (Esser & Haider, 2017) also could be the target of metacognitive learning processes. Thus, it is open to further investigation whether such a higher-order process would also be able to learn about different metacognitive judgements (e.g. fluency). The mechanism described by Cleeremans et al. (Pasquali et al., 2010) has so far only been tested in situations where a person is directly asked to evaluate the correctness of their responses. This leads to the question whether metacognitive learning only occurs for intentionally attended dimensions (e.g. correctness), or whether learning about first-order performance is automatic and can happen in parallel for multiple dimensions (e.g. correctness, fluency, speed, etc.) when there is no external instruction to do so (as there is by subsequently presenting the post-decision wagering task).

Research on implicit learning implies that implicit learning processes can occur in parallel (Goschke & Bolte, 2012; Haider et al., 2012, 2014, 2020; Mayr, 1996). Yet, it is not granted that higher-order learning processes can happen in parallel for all implicit learning processes. It might be that higher-order learning processes rely on intention, respectively selective attention, to evaluate one specific behavioral output (correctness, speed, fluency, etc.). If this were the case, it needed to be explained how the system decides which first-order representations are accessed to develop a higher-order representation. Moreover, a large number of the implicit learning studies cited here, imply sensitivity for sudden changes, which so far have not been addressed by theories suggesting gradual metacognitive learning processes.

Therefore, a learning process involving expectations, predictions, and violations thereof should be considered rather than gradual associative strengthening. On an empirical side, this is supported by the above-mentioned studies, which used different manipulations for balancing the associative strength between conditions but manipulated whether small or large violations of expectations occurred. For example, Esser and Haider (2017) showed differences in the emergence of explicit knowledge when the structure of the task led to noticeable differences in the fluency of processing the task material. Noticeably, the number of regular and irregular sequential trials was equal for both groups. Therefore, it needs to be addressed how a metacognitive mechanism that gradually learns to evaluate first-order performance would detect the differences between both learning conditions, even though the first-order signal strength is matched. A gradual bottom-up higher-order learning mechanism does not include the size of prediction errors (here, the sudden changes in fluency) as a signal. In the following section, we will propose a tentative model which includes ideas of the Higher-Order Thought Theories and the Global Workspace Theory to respond to the formerly described problems.

## Metacognitive learning mechanisms and unexpected events

**Fig. 1:**
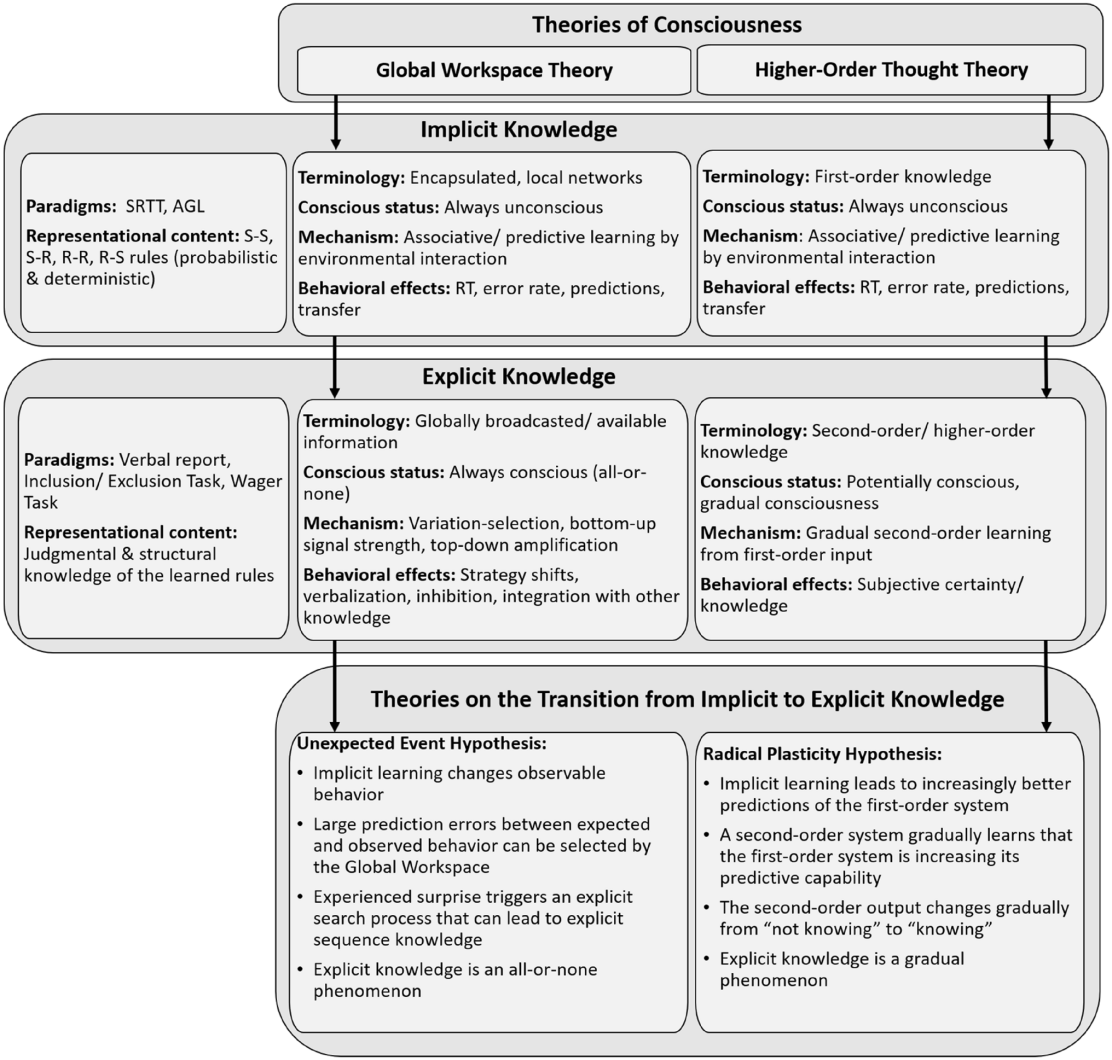
Implicit and explicit learning viewed under the Global Workspace and the Higher-Order Thought Theory

We have reviewed two different views on the development of explicit knowledge in an implicit learning situation: The Unexpected Event Hypothesis (Frensch et al., 2003; Haider & Frensch, 2005) and the metacognitive Radical Plasticity Hypothesis from Cleeremans (2008, 2011). We have argued why the Unexpected Event Theory makes assumptions and provides empirical evidence that fits with a Global Workspace Theory of consciousness, while the Radical Plasticity account of Cleeremans theoretically and empirically fits well with Higher-Order Thought Theories (see Fig. 1).

First, both theoretical viewpoints do not differ in the conceptualization of the process of implicit learning itself. Implicit learning is viewed as a first-order learning mechanism (as it might be called under a Higher-Order Thought Theory viewpoint), that creates localized, encapsulated representations (as the Global Workspace Theory would put it). These first-order learning processes can be described as internal perception–action loops that use prediction errors to enable learning. In such models, learning of actions or action-sequences is controlled by an interaction of feedback and feedforward loops (McNamee, & Wolpert, 2019; Wolpert & Ghahramani, 2000). A forward model predicts, given a specific motor command, sensory or proprioceptive consequences of an action. The predictive forward model can be trained by comparing predicted and actual sensory feedback and using the resulting error signal to make increasingly more accurate predictions. It has been demonstrated that such internal perception–action loops are relevant for implicit motor and perceptual sequence learning (Janacsek et al., 2020; Lutz et al., 2018; Ruttle et al., 2021; Ziessler & Nattkemper, 2001).

Both theoretical viewpoints, the Unexpected Event Hypothesis and the Radical Plasticity Hypothesis state that it is not these implicit internal models themselves that are consciously accessible. Instead, both theoretical approaches raise the question how a secondary explicit or higher-order learning mechanism represents that the contents of the first-order models have changed and, finally, how consciousness about unconsciously learned sequences arises.

Both viewpoints have their strengths and weaknesses. The metacognitive Radical Plasticity account has the strength of explaining conscious access by clearly definable connectionist learning mechanisms that rely on the same predictive learning principles as the first-order system does. However, this does not readily explain how structural explicit knowledge develops (knowing what exactly the sequence is; Dienes & Scott, 2005). Furthermore, it does not account for the role of expectancy violations from several distinct metacognitive sources (accuracy, speed, fluency, conflict, etc.).

The seemingly biggest difference between the Radical Plasticity account and the Unexpected Event Hypothesis is that the former assumes that consciousness develops gradually along with lowering the prediction error of the second-order system; when there is no surprise about the ongoings in the first-order system left, one knows that one knows. The Unexpected Event Hypothesis instead proposes that conscious awareness is triggered by large prediction errors; when an individual thought they did not know but apparently know. The Unexpected Event Theory captures the important aspect of violations of consciously accessible expectations and explains why a rather sudden insight (comprising the time span from a consciously accessible surprise to explicit structural knowledge) into the implicitly learned representations seems to develop. Its weakness is that, so far, it did not take metacognitive learning mechanisms into account that could provide a clearer prediction when expectancies will be violated or how these expectancies arise in the first place.

Thus, we aim to elaborate the processes behind the original proposal of the Unexpected Event Theory and to point to open questions which should be addressed by future research. Even though the Unexpected Event Hypothesis generally follows the Global Workspace Theory, we assume that higher-order learning is an important mechanism to consider, when trying to explain how an unexpected event can become consciously accessible. What is needed is a mechanism which allows a comparison between the expected metacognition (e.g., “How correct, fast, fluent, (...) should my response be?”) an individual has in a given situation and the experienced metacognition (e.g., “How correct, fast, fluent, (...) was my response?”). Important questions in this regard are: what is the relation between the first-order internal perception–action models and the second-order or metacognitive model? How could a metacognitive learning process explain how a consciously accessible surprise occurs? Currently, there are several different models aiming to explain the relation between the first-order signal (here, implicit knowledge) and the second-order metacognitive evaluation of these signals. Mostly, these models rely on signal-detection theory (Del Cul et al., 2009; Galvin et al., 2003; Lau & Rosenthal, 2011). The problem with these models is that they are often pure bottom-up models that do not take important top-down factors into account which have shown to influence metacognitive decisions. This includes, for example, the use of heuristic cues (e.g. fluency, luminance), which have no direct relation to the first-order signal the metacognitive judgement is relating to (Hoyndorf & Haider, 2009; Koriat, 2007; Wilbert & Haider, 2012). It further includes the role of previous metacognitive experiences, with similar situations, successes and failures, or general knowledge about one’s own performance capacities.

There are, however, theories that model the relationship between first-order knowledge and metacognitive judgements with Bayesian learning (Fleming & Daw, 2017; Sherman et al., 2015). One advantage is that Bayesian models allow metacognitive learning via predictive coding (Clark, 2013; Friston, 2010). The evaluation of one’s own behavior, respectively knowledge leads to a first hypothesis of what metacognitive experience is expected in the next, similar situation. This prediction is compared with the current experienced metacognitive judgement and, in turn, the resulting error-signal is used as a bottom-up learning signal for the next, more precise metacognitive prediction.

For implicit learning and the development of explicit knowledge, this means that any individual has a certain expectation about their own performance in an SRTT, based on previous experiences with similar situations. Thus, in the beginning an individual has a certain metacognitive model how fast, fluent, correct, etc. their behavior should be when responding to stimuli that appear seemingly randomly on a computer screen. The sequential material inherent in the task can lead to behavior different from the expected behavior. These deviations from the expected performance will be used to adjust the metacognitive model. To develop explicit knowledge, the participant has to recognize that their performance does not match their expectations to an extent that is not compatible with their model of the current situation. We assume that what is important for a change of the metacognitive model and thereby the development of explicit knowledge, is the size of the metacognitive prediction error and the strength of the a-priori hypothesis*.*

Smaller deviations from the expected metacognitive judgement of the situation can easily be used to adjust the model via this bottom-up error signal. For example, faster responses, fewer errors, and increasing fluency are compatible with mere practice effects and only slight, gradual adjustments of the metacognitive models are the result. However, large prediction errors would more likely lead to a stronger change of the metacognitive model. In this case, it might be functional to evaluate whether a new, different model should be applied to the situation, instead of making rather drastic changes to the current model. The strength of the a-priori hypothesis could also play a significant role. If there is a very strong a-priori hypothesis and the current data strongly contradict this model, it might be less functional to make drastic changes to the current, well-established model. Instead, it might be more advisable to test whether a different model should be applied, respectively built for the new situation. During an SRTT-training, a large deviation might occur, if the well-practiced sequence is suddenly removed and replaced by random responses. In this case, it might be less functional to assume that the own performance capacity has declined and instead it might be useful to check whether the task has properties that were not considered before (e.g. that there used to be a sequence, which is now missing). If, however large metacognitive prediction errors are encountered while the participant only has a rather weak a-priori hypothesis, there is no need to replace the current metacognitive model with a new one. Instead, the participant will make adjustments to their expectations, without developing explicit sequence knowledge. Taken together, we propose that both factors, metacognitive prediction error and strength of the metacognitive a-priori hypothesis determine whether an individual will change their metacognitive model and thus enable explicit sequence learning.

As mentioned before, there are models for metacognitive learning that support our notion that metacognitive evaluations not only rely on current first-order performance signals but also on earlier metacognitive judgements (e.g. Fleming & Daw, 2017). So far, there is not much research on how metacognitive models are selected in a given situation and under which circumstances a model is replaced with a new or different one or when instead the current model will be adjusted. Collins and Frank (2013) suggested a Bayesian “context-task set” model. In this model, an inference is made in every single learning trial about whether the current task-set is still applicable to the current situation or whether there are yet unknown rules that should influence the task-set and, therefore, a new model should be applied. This model also uses arbitrary context cues to determine whether the current situation is indicating a new, unknown task context or whether previously acquired metacognitive models should be used and adjusted.

Importantly, with regard to the Global Workspace Theory, we do not assume that such metacognitive representations are per se conscious or that their strength has any relation to a gradual change in consciousness. Instead, we propose that metacognitive representations are generally good candidates for being accessed by the global workspace (see Shea & Frith, 2019, for an opinion why metacognitive learning is important to the Global Workspace Theory). Assuming that there are multiple parallel higher-order learning processes, their content might be entirely implicit as well. They send and receive information from the global workspace, just like implicit first-order information could. In the global workspace, multiple meta-cognitive representations could be integrated into a hierarchically higher metacognitive representation of the current situation. This integrated information could for example involve metacognitive knowledge about one’s own performance like fluency, accuracy of processing, precision or confidence. In addition, different weights to the underlying metacognitive representations are assigned, according to their current relevance.

These original assumptions of the Unexpected Event Theory and the additional assumptions about a predictive metacognitive learning processes proposed here, could solve some of the formerly described problems behind the explanations based solely on the Global Workspace Theory or Higher-Order Thought Theories. Concerning the Global Workspace Theory, the Unexpected Event Theory does not need to explain how an implicitly acquired first-order representation can gain a signal-strength strong enough to win the competition against all other unconscious modules or how top-down amplification can be directed to this encapsulated knowledge. This problem is solved because it is the conflict between the expected and the experienced metacognitive judgements which gains access to the global workspace. It is the representation of this conflict (or surprise) which has a high likelihood of winning the competition against other parallel processes for entering the global workspace.

Concerning the Higher-Order Thought Theory-based explanation of the emergence of explicit from implicit knowledge, the here proposed addition to the Unexpected Event Theory account lies in the assumption how implicit, first-order knowledge and higher-order knowledge are related. An account where metacognitive judgements depend on a predictive learning process that does not only base its predictions on the first-order bottom-up signal, but also on heuristic cues, previous knowledge, and experiences with similar situations, can help to explain different empirical findings. This includes, for example, premature responses (Haider & Frensch, 2009), changes in the underlying sequence (Schwager et al., 2012) and changes in the experienced fluency (Esser & Haider, 2017; Rünger & Frensch, 2008). All these results are difficult to explain with a pure bottom-up mechanism relying on gradual strengthening of the first-order learning signal. Furthermore, large prediction errors and the processes, they are assumed to trigger, fit the data suggesting that explicit knowledge seems to develop in a sudden moment of insight (Haider et al., 2011; Rose et al., 2010; Schwager et al., 2012; Wessel et al., 2012), rather than developing gradually.

## Conclusion and future directions

We assume that the predictive metacognitive model a person has about their own behavior in a given situation (e.g. how fast, how precise, how difficult or fluent a task should be) adapts to the task by comparing the predicted and the experienced metacognitive judgement in any given situation. The behavioral changes resulting from implicit learning may not fit the current metacognitive model (i.e. responses might suddenly be much slower than expected when the sequence is exchanged with new, random material). If so, this violation has a high chance to enter the Global Workspace and serve as a trigger to evaluate whether a new metacognitive model of the situation should be applied.

Nevertheless, there are questions that should be addressed by future considerations. This relates to theoretical assumptions in need of further elaboration: Are all implicit learning processes continuously monitored by parallel metacognitive learning processes? How can previous knowledge and external cues, like task fluency, influence these metacognitive processes? Is metacognitive learning a pure bottom-up process? Does the metacognitive prediction error play a role in the emergence of explicit knowledge? Furthermore, there is a need for a model that can account for current empirical findings: Why does explicit knowledge seem to emerge in a sudden insight process? Why do alternative explanations for the behavioral change prevent such insight?

We assume that the Unexpected Event Theory and the here proposed extensions about expectancy violations resulting from Bayesian metacognitive learning processes can provide a promising step into answering these questions. The prediction of a metacognitive judgement is compared to the currently experienced metacognitive judgement about one’s own behavior. Its prediction-error signal is then used as a learning signal for developing a more accurate metacognitive model of the current situation. Small prediction errors might lead to a gradual change of the model. Yet, large prediction errors in combination with strong a-priori hypotheses can serve as a signal that the current model is not suitable for the given situation and a different model should be applied. Within such a framework, it can be modelled that not only the current bottom-up first-order signal but also top-down factors, such as heuristic cues and previous experiences with similar situations, are the basis for a prediction of the metacognitive judgement in a given situation.

Our proposal of integrating the role of metacognitive learning processes in the Unexpected Event Theory needs further experimental investigation: First, it should be tested whether the predicted metacognitive judgements can be manipulated not only by the strength of the first-order signal but also by differences between the expected and the actual experienced metacognitive judgment. Second, the size of the prediction error of metacognitive judgements as well as the strength of the a-priori hypothesis should be manipulated to test its relation to the emergence of explicit knowledge. Third, we proposed that large prediction errors serve as a consciously accessible signal to trigger explicit search processes. These search processes are assumed to lead to a new explicit representation, independent of the implicit representation.

A better understanding of the transition from implicit to explicit sequence knowledge can provide interesting contributions to the broad and difficult field of consciousness theories itself. Implicit learning paradigms create the unique experimental situation where unconscious knowledge does not need to be induced by week signal strength or inattention. The development of metacognitive knowledge is a concern of many different and often separated research fields which all provide different contributions. For example, research on decision-making or on perception is governed by bottom-up signal-detection models (Galvin et al., 2003; Pleskac & Busemeyer, 2010), cue-utilization is prominent in memory research (Koriat, 2000, 2012, 2015) and models of evidence accumulation are often found in research on error-monitoring (Yeung & Summerfield, 2012). Implicit sequence learning paradigms can augment this research by providing additional opportunities (to the predominant priming paradigms) to manipulate the first-order signal strength, external cues, as well as the role of prior expectations and how these expectations develop over the course of learning.
